# A communication-focused curriculum for dental students – an experiential training approach

**DOI:** 10.1186/s12909-018-1174-6

**Published:** 2018-03-27

**Authors:** Simone Alvarez, Jobst-Hendrik Schultz

**Affiliations:** Department of Internal Medicine and Psychosomatics, University Hospital Heidelberg, Heidelberg, Germany

**Keywords:** Experiential learning, Self-awareness, Dental studies, Curriculum development, Communication

## Abstract

**Background:**

Successful interaction and communication with patients is as vital for dentists as it is for physicians. Therefore, the aim of this study was the development and evaluation of an interactive, experiential training curriculum with an emphasis on communication for dual degree seekers of medicine and dentistry.

**Methods:**

A pre-clinical course with an emphasis in physician/dentist-patient communication and interaction was adapted by a team of subject experts based largely on the propositions of Experiential Learning Theory. After attending the course, dental students (*N* = 81) rated the course on a Likert- style scale and answered two open questions.

**Results:**

Students found the interactive course curriculum to be very helpful and vital. Many students reported that their initial interest in the course was mainly because it is a dual degree requirement, but later on rated the course as highly important in terms of later physician/dentist-patient interaction. One aspect of the curriculum course participants regarded as very important, yet neglected during dental studies, was (self-) perception.

**Conclusion:**

In the view of dental students, the rigorous structure of the pre-clinical dental curriculum does not allow for time spent on topics such as (self-) perception and awareness, but training one’s ability to self-reflect and think critically about one’s own actions, conduct or position can aid with advanced medical and dental studies and practices later on. Experiential courses with an emphasis on patient-physician/dentist communication should be offered early on during pre-clinical medical and dental studies as a regular part of the curriculum.

## Background

Effective physician-patient communication skills are as important to medical care as clinical expertise [1, 2]. The same is true for dentist-patient communication [3–7]. It is known that good physician/dentist-patient communication can lead to a reduction in patient anxiety, and increase factors such as patient satisfaction and adherence to healthy behaviors [8, 9].

Thus, successful physician/dentist-patient communication is increasingly becoming a central learning objective at medical schools in Germany. Competency frameworks such as the German National Competence-based Learning Objective Catalogue for Undergraduate Medical Education (NKLM) and the German National Competence-based Learning Objective Catalogue for Undergraduate Dental Education (NKLZ) have been designed to ensure quality standards in regard to medical and dental education. Within this framework, communication is described as a main competency [10]. To ensure the necessary time to practice skills, many schools have established courses with an emphasis on communication [6, 11–13].

At the Medical Faculty of Ruprecht Karls University Heidelberg, Germany, medical students take part in a longitudinal communication training that also encompasses training with simulated patients [14]. From the very first semester onward during the pre-clinical phase up until the end of the clinical phase of studies, students are required to attend courses, seminars and tutorials with a focus on physician-patient communication. However, communication training for dental students usually does not start until the clinical phase of studies. One opportunity for dental students to attend dentist/physician communication training during the pre-clinical phase of studies is if they are planning to obtain a dual degree of dentistry and medicine. In that case, dual degree seekers have to attend courses and seminars in medical psychology and sociology in addition to their regular class load. One major concern of these seminars and courses and sociology is the teaching of successful patient-centered communication.

Within the context of the current amendment of study regulations at this medical school, the authors of this article argue that it is crucial that students of dentistry should also receive the opportunity to attend some type of training with a focus on communication early on during the pre-clinical part of dental studies. After all, the many benefits of a course curriculum that focuses on soft skills such as communication and self-awareness have been established in the past [15], and the current literature provides much information on the positive effect of structured communication training on dental students’ communication skills [13, 16, 17]. Some courses may even have the potential to influence students’ attitudes towards the subject [11].

Research also indicates that during the course of their studies, medical and dental students show an increase of psychosocial stress due to the demanding nature of the training [18–20]. In order to keep stress for students to a minimum during the pre-clinical phase of studies, a course in medical psychology with an emphasis on physician-patient communication was adapted on the notion that students could learn the content mostly in an experiential way. The goal was the assembly of a curriculum that would be structured, realistic and applicable, but first and foremost: very interactive. It was to provide a good complement to the more structured, lecture-style courses that make up the majority of pre-clinical studies. During this course, students were to have the opportunity to learn through experiment and experience, which raises the possibility of knowledge acquisition and retention during class and keeps study time outside of class to a minimum. Based on the results of previous research [11], it was hypothesized that by giving only brief theoretical input and then encouraging students to “simply give it a go” with actual hands-on exercise, an awareness of the importance of the subject (physician/patient-communication) could be developed, and student motivation towards the subject as well as student perception of its importance, influenced.

The focus of this study is a course of medical psychology and sociology for dual degree seekers which has been in place for two consecutive academic years (2014–2015) and consists of 20 teaching units, spread out over four sessions during two weekends. The course is offered three times during the academic year with an average group size of 12–14 students, and attending students are usually in their third semester of dental studies.

### Course curriculum development

Student-centered teaching approaches have become increasingly common in medical education. For example, during problem-based learning (PBL) style courses, students are actively challenged to learn through engagement in real-world tasks and problems. This approach usually begins with the presentation of a medical problem for which students have to find a solution by using problem-solving strategies. Instructional guidance is kept to a minimum. Previous studies on this approach show that students very much appreciate the opportunity to work on “real” problems and that it facilitates acquisition of skills and ensures retention of knowledge [21]. A less investigated teaching approach is Experiential Learning theory (ELT). Just as PBL, ELT is a very interactive method of learning, and it is based on the premise that learning is the process of creating knowledge, and that during this process, the learner goes through the following four stages in a recursive process: experiencing, reflecting, thinking, and acting. Each stage is responsive to the individual learning situation and the content learned [22].

This course described in this article was adapted by two experts in the field of medical education based on the propositions of ELT. The goal was to design a flexible curriculum that could easily be adapted according to individual progress of each group and the overall pace of learning in which groups naturally differ. The specific course structure was then determined following the seven principles for efficient teaching by Hannah et al [11]. Table 1 illustrates these strategies and the way in which each one was incorporated into the curriculum.

**Table 1:** Application of the seven teaching principals

Principal	Application
Use a skills-based approach, as opposed to an exclusively didactic approach	Exercises during which students were engaged and challenged directly were included
Use clinically relevant scenarios	Scenarios that were relevant to both, dentists and physicians were included
Allow for self-assessment by students	Students were encouraged to assess their own performance based on videos filmed during exercises
Use videotaping method	This method was used mainly for the purpose of self-reflection and –assessment
Use simulated patients with expertise in a variety of clinical roles and in the monitoring of student performance and the delivery of feedback	Simulation patients were used specifically for feedback purposes and to give students the opportunity to experiment with the various communication techniques learned during the course
Use an integrated teaching team comprising health sciences staff and human sciences disciplines	The course curriculum was developed by a team of medical and psychological experts in the field of communication; however, the course was taught by one person only, a psychologist
Ensure small groups for optimal student learning	Maximum group size was at 14 participants

The outcome was a structural blend of elements such as specific communication techniques, communication exercises with simulation patients (SP), analysis of video films, and exercises of (self-) awareness and (self-) perception. During the course, students were to not only practice their active listening skills at the example of medical history taking, but also to receive the chance for practical application during discussions, practical exercises, and group/team work. Stimulating communication among each other and with the instructor, paired with interesting content and cooperative learning structures were to motivate students to engage in a joint thinking and learning process, and thus promote multiple points of view. Content was to be delivered in a vivid and engaging way, so a high presence of cooperative methods such as group work, as well as activity-oriented methods such as simulation, role play, or case studies were included. These methods also allow for instructor and peers to give feedback in a way that would directly influence the students need to be rewarded, and thus cause positive emotions and a feeling of accomplishment [23]. Direct frontal instructor input was restricted to the absolute minimum. Table 2 illustrates the main components of this course curriculum.

**Table 2:** Curriculum – Communication training for dental students

Subject	Learning objective(s)	Instructional methods	Approximate time spent on task (in hours)
Biopsychosocial Model	Learn about the importance of communication for successful physician-patient interaction and the healing process	Brief instructor input Self-reflection Group discussion	2
Communication techniques and strategies	Learn about and practice active listening at the example of medical history taking	Brief instructor input Practical exercises Practical exercise with SP	4
(Self-) awareness: external and (self) perception	Become aware of “self” Understand the impact of internal subjectivity in one’s attitudes, values, and beliefs of reality Recognize one’s feelings during “easy” or “difficult” patient-physician interactions	Brief instructor input Visual Thinking Strategy Role play Practical exercise with SP	5
Communicating empathy and emotional awareness/understanding	Learn how to use means of non-verbal communication effectively Understand the difference between feelings of sympathy and feelings of empathy	Brief instructor input Video film analysis Group discussion Reflection	3
Attitude, bias and emotion-handling	Learn how personal attitudes, biases, fears, emotional reflexes, psychosocial defenses, and moods can interfere with one’s ability to arrive at an accurate diagnosis, prescribe appropriate treatment, and promote healing	Brief instructor input Prompting questions Group discussion	3
Personal development and well-being	Become aware of the importance of personal development and well-being Recognize personal limits and learn when to seek support and consultation	Brief instructor input Group discussion	3

## Methods

### Participants and procedure

After having attended the last day of the course, all dental students (*N* = 81) who had participated were asked to fill out a survey which consisted of ten standardized Likert- scale items and two open questions. Students were given about 20 min to fill out the survey and were verbally encouraged to take time for adequate reflection on the individual questions.

### Survey

Students had to rate ten items pertaining to the course they had attended in Likert type scale (Table 3). Additionally, two open questions asking students to name the aspects they liked about the course and also the aspects they thought could be improved. Return rate of the survey was 100%. Data were analyzed using SPSS statistics software. The open questions were evaluated according to qualitative content analysis and frequency analysis with the qualitative analysis software program MAXQDA [24].

**Table 3:** Survey results

Item	M	SD
1.) How much interest did you have initially to participate in the course?	1.69	.79
2.) Did the course increase your interest in the subject matter?	1.46	.79
3.) Has the course helped you develop more confidence in your communication skills?	1.69	.79
4.) How helpful did you find the practical exercises?	1.59	.76
5.) How much have you learned about patient-centered communication?	1.68	.83
6.) How helpful did you find the exercises with simulation patients?	1.35	.55
7.) How relevant would you rate this course for a later physician/dentist – patient interaction?	1.65	.79
8.) Was the trainer sensitive to student needs and concerns?	1.15	.53
9.) Was a good balance of student participation and trainer contribution achieved?	1.19	.55
10.) On a scale from 1 (very good) to 5 (very bad), how would you rate the course overall?	1.69	.69

Scale: 1 (very much) to 5 (not much at all); *M* mean, *SD* standard deviation

## Results

### Quantitative results

The survey results in Table 3 show that many students reported that their initial interest in the course was due to the fact that it is a dual degree requirement.

However, later on students rated the course as highly important in terms of physician/dentist-patient interaction. Quantitative analysis revealed that many students already had an initial interest in the subject, but also that this interest had increased during or after the course. Students even considered the training of communication skills to be more important after they had attended the course than prior to it. Specific aspects of the curriculum course participants regarded as especially important were (self-) awareness and (self-) perception. The practice of communication skills with teacher-, peer- and SP feedback and a particular focus on self-perception and –awareness were valued highly by students because it provided a different learning experience in a curriculum that is sometimes perceived as rigid. Especially the opportunity to experiment with what has been learned, and time to practice in the safety of a simulated situation during role play and SP exercises were very much appreciated.

### Qualitative results

Content analysis of the open questions showed that students experienced the course to be very applicable and useful in terms of a later dentist/physician-patient interaction (25 indications). One aspect that seemed to have left a positive impression was that the course was viewed as very interactive. The ratio between instructor input and active student participation was experienced as stimulating and within this context, the multitude of different teaching methods led students to feel especially involved with the course content. The opportunity to practice the various techniques introduced right away with simulation patients was named as very helpful because it allowed for experimentation, personal experience and reflection. Also, the importance of the role and qualities of the instructor became apparent. Frequently, the instructor was named crucial for the creation of a pleasant learning atmosphere and the perceived flexibility of the course. The subject of awareness was well-received, since according to students’ comments in the qualitative section of the questionnaire, active reflection and self-analysis was barely possible anywhere else during pre-clinical studies. Especially the opportunity to readily try out what has been learned without worrying about negative judgment has been valued by course participants. Table 4 illustrates the frequency with which the indications have been made as well as the codes on the positive spectrum which have been constructed using an inductive approach [24].

**Table 4:** Code system – positive spectrum

Code	Number of indications
Applicability of course content	25
Interactive nature of the course	23
Variety of teaching methods	19
Instructor qualities	16
Course atmosphere	16
Course flexibility	15
Opportunity to practice with SP	13

Critique of the course was largely limited to organizational aspects. In the view of students, weekend courses pose an extra challenge in an already rigorous semester. Even with the course being an add-on to regular dental studies, some students wished that it would have been part of the regular curriculum. Some thought that the relevancy of the course would have been even higher during clinical studies, because it would have allowed for immediate practice with real patients. Some would have liked to have had more time for the individual exercises, especially for the ones with simulation patients. One major criticism was that because of time constraint not all students received the opportunity to practice with a simulation patient. Table 5 illustrates the coding of the negative spectrum.

**Table 5:** Code system – negative spectrum

Code	Number of indications
Time frame of the course is too short	23
Weekend sessions are inconvenient	19
Course would fit better during clinical part of studies	8
Not everyone was able to practice with SP	6
Course is not part of the regular dental curriculum	6
Individually shorter, but more frequent sessions	6

## Discussion

Previous research has demonstrated that medical students have positive attitudes towards learning and using communication skills [25–27] and within this context, experiential methods have been shown to be more effective in teaching communication skills than lecture methods [11, 12]. The present study confirms previous findings by demonstrating that experiential learning activities allow students to apply abstract concepts in realistic patient-care scenario and that the use of experiential methods may help medical students to appreciate the importance of learning communication skills [11, 28, 29]. In the described course curriculum, brief theoretical elements were combined with a multitude of practical exercises. Through this active, social learning process, students received the possibility to explore their own concepts, attitudes and values. Even though some students appeared to have needed an initial phase of “warming-up” to the experiential approach, methods such as role play and simulation exercises with SP were evaluated positively because it provided them with feelings of competency, autonomy, and social inclusion; aspects that can cause students to act intrinsically as opposed to simply acting according to a standardized procedure [30]. Positive experience, identification, and the analysis of personal emotions are vital for learning and motivation so during the course, aspects of (self-) perception were emphasized. From the perspective of educational psychology, the practical exercises used during the course also increased the likelihood of reception and retention of new knowledge [22].

Overall, experiential learning seems to be a valid alternative to structured education based curricula. However, as a curriculum designer and as an instructor, one has to be willing to accept that this approach at times might be flawed in a technical aspect, and that not all students are comfortable with the perceived lack of guidelines, but it does have the potential to awaken unknown motivation for the subject in students. In general, the present study showed that the subject of physician-patient communication is experienced by students as an important one. In particular, it was found that the experimentation with, and the reflection on the experience have been rated as particularly helpful by students and also had a positive influence on their attitude towards the subject.

## Conclusion

In the fields of dentistry and medicine, knowledge and technical skills are not sole determinants of good practice. Active listening skills, gathering and communicating information effectively, handling patient emotions sensitively, demonstrating empathy, and awareness are also crucial aspects. In this study, it has been demonstrated that an experiential learning approach can be used to motivate students, influence their perception of the subject of communication, and provide opportunity for self-reflection and awareness. Thus, dental students should be given the opportunity to learn about, experiment with, and reflect on their communication strategies early on during their studies, so that they may be able to successfully communicate with patients later on.

## Abbreviations

ELT: Experiential Learning Theory
SP: Simulated patient
